# Metabolomics-Based Study: Effects of Fructose, Glucose, and Mannose on Islet Cells and Change of Serum Markers in Streptozotocin-Induced Diabetic Rats

**DOI:** 10.2174/0115733998394170251129101618

**Published:** 2026-02-13

**Authors:** Shuang Guo, Tao Liu, Rong Li, Lijun Zhang, Xueyan Li, Xiaomei Long, Fangfang Chen, Jianxing Gu, Yuan Fan, Zhusheng Tang

**Affiliations:** 1 Yunnan University of Traditional Chinese Medicine, Kunming, Yunnan, 650500, China;; 2 Tianjin University of Traditional Chinese Medicine, Tianjin, 301617, China;; 3 Yunnan Provincial Hospital of Traditional Chinese Medicine, Kunming, Yunnan, 650021, China;; 4 Baoshan Hospital of Traditional Chinese Medicine, Baoshan, Yunnan, 678000, China;; 5 The Second Affiliated Hospital of Yunnan University of Traditional Chinese Medicine, Yunnan, 650500, China

**Keywords:** Fructose, glucose, mannose, streptozotocin-induced rat, serum metabolism, islets cells

## Abstract

**Introduction:**

Experiment uses metabolomics to explore the effects of glucose, fructose, and mannose on the metabolism and pancreatic islet structure of rats, providing ideas and references for clinical and practical applications.

**Method:**

In this experiment, rats were randomly divided into four groups of 10 rats each: the normal control group, glucose group, fructose group, and mannose group. The sugar for each group was dissolved in water and freely ingested by the rats for 8 weeks. After 8 weeks, 5 rats were randomly selected from each group and injected intraperitoneally with STZ. After three days of observation, tissues were collected. Pathological tissue staining was performed to analyze the structure of rat pancreatic islets, and metabolomics analysis was conducted on rat serum. Based on the differential metabolites, predictive analysis of the metabolic pathways of the three monosaccharides was performed.

**Result:**

The results showed that glucose had a protective effect on rat pancreatic islet structure and reduced the degree of islet destruction caused by STZ. Fructose had a weaker protective effect than glucose but still demonstrated some protection. Mannose showed almost no protective effect on rat pancreatic islet function.

**Discussion:**

Serum metabolomics analysis suggested that the possible mechanisms underlying the effects of different monosaccharides on rats involved tyrosine metabolism, pentose phosphate metabolism, and Type 2 diabetes mellitus. In contrast, the potential mechanisms associated with STZ injection were related to arginine and proline metabolism, the mTOR signaling pathway, and amoebiasis.

**Conclusion:**

This experiment systematically revealed the differential effects of glucose, fructose, and mannose on the structural protection and metabolic pathways of rat pancreatic islets through metabolomics and histopathological analyses, providing an important theoretical basis for diabetic nutritional intervention and drug target development.

## 1 INTRODUCTION

Fructose is one of the most common hexose monosaccharides. It is usually found in its free form in common foods such as honey and fruits. Studies have shown that fructose is metabolized in the body through the production of fructose 1-phosphate, catalyzed by fructokinase, mainly in the liver, kidney, and small intestine [1]. It may also be converted to glucose or synthesized into glycogen *in vivo* under the catalytic action of enzymes. The metabolism of fructose in the body is mainly concentration-dependent, and less insulin-dependent [2]. However, fructose is converted *in vivo* and affects the enzyme fructose-1,6-diphosphatase, which, in turn, affects insulin resistance [3]. Studies have shown that high fructose diets significantly increase the risk of visceral fat accumulation and hepatic insulin resistance, one of the central triggers of metabolic syndrome [4, 5]. Glucose is one of the most widely distributed and important monosaccharides in nature, and one of the main components of the body's energy source and metabolism. Some studies have shown that short-term intake of glucose can protect the kidneys and reduce the effects of ischemia-reperfusion injury on renal tissue [6]. After entering the body, glucose is broken down by enzymes and enters the bloodstream, where it participates in energy-supplying reactions that provide energy to the body. Glucose metabolism disorders are associated with the occurrence and development of diseases, such as glucose metabolism and myocardial energy metabolism.They are closely related, and regulating glucose metabolism has become one of the effective treatments to improve myocardial ischemiareperfusion injury [7]. Beyond that, improving glucose metabolism may be beneficial in Parkinson's disease and may play a role in diagnosis and treatment [8].

Mannose is a monosaccharide found free in the pericarp of certain plants. As the only clinically applied glyconutrient, it is widely distributed in one’s body fluids and tissues and is directly involved in protein synthesis and the regulation of the immune system [9]. There is an association between glucose metabolism and glycemic changes, but no studies have demonstrated a mechanism for this association [10]. Relevant studies have shown that mannose has significant anticancer effects [11], as well as the ability to interact with E. coli and influence the development of diseases such as urinary tract infections [12].

Diabetes is a globally prevalent chronic disease, with approximately 463 million adults worldwide living with diabetes as of 2021, and this number is expected to increase to 700 million by 2045, according to the International Diabetes Federation (IDF) [13]. It is mainly classified into Type 1 Diabetes Mellitus (T1DM), Type 2 Diabetes Mellitus (T2DM), Gestational Diabetes Mellitus (GDM), and other specific types. T1DM is common in children and adolescents [14] and manifests as insufficient insulin secretion, requiring lifelong insulin therapy. T2DM, which is most commonly seen in middle-aged and elderly people, has increased in prevalence among young people in recent years. It is caused by insulin resistance and insufficient secretion. It is treated by lifestyle intervention and pharmacological therapy. T2DM is caused by insulin resistance and insufficient secretion, and its treatment mainly includes lifestyle intervention and drug therapy. Some studies have shown that the intake of desserts or sugary drinks has an impact on people's health, affecting the body's metabolic functions, which can lead to weight gain and even the risk of developing diabetes [15].

The experimental design used glucose, fructose, and mannose interventions combined with STZ injections, with the aim of providing a molecular mechanism reference for dietary interventions in diabetes and promoting research on the clinical application of functional monosaccharides.

## 2 METHODS

### 2.1 Laboratory Materials and Equipment

Streptozotocin (STZ), purity > 98%, was purchased from the Shanghai Macklin Biochemical Co., Ltd.; glucose, fructose, and mannose were purchased from Shandong Xiwang Pharmaceutical Co. Mass Spectrometer (Thermo Scientific, USA, Item No. Q Exactive Plus), UHPLC (Shimadzu, China, Item No. Nexera X2 LC-30AD), Chromatography Column (Waters, USA, Item No. ACQUITY UPLC BEH Amide 1.7 μm, 2.1mm × 100 mm column), Ultrasonic System (Diagenode, USA, Article No. Bioruptor), Vacuum Centrifuge Concentrator (Eppendorf, Germany, Article No. Concentrator), Centrifuge (Eppendorf Centrifuge, Germany, Article No. 5430R), Visible UV Spectrophotometer (Thermo, USA, Article No. Bioruptor), and Photometer (Thermo, USA, item no. MultiSkan FC).

### 2.2 Animal Model

Eighty Wistar rats, 48 weeks, male (180-200 grams), were purchased by Yunnan Commitment Technology Co Ltd, Licence (SCXK (jing) 2019-0010), and kept in the Animal Experiment Center of Yunnan University of Traditional Chinese Medicine. The animal experimental research program has been approved by the Animal Ethics Committee of Yunnan University of Traditional Chinese Medicine (Approval No. R-062021G088).

The rats were randomly divided into 4 groups (n = 10): control, glucose, fructose, and mannose. The rats were given drinking water, 20% glucose water, 20% fructose water, and 20% mannose water, respectively, and were allowed to drink ad libitum for 8 weeks. After 8 weeks, the rats in each group were randomly divided into two subgroups, creating a total of eight groups (n = 5). The rats in the new subgroups were injected intramuscularly with STZ at 35 mg/kg, and their blood glucose levels were monitored for three consecutive days.

### 2.3 Metabolomics Testing

100 μL of rat serum was mixed thoroughly with 400 μL of cold methanol/acetonitrile (1:1, v/v) by vortexing. The mixture was then sonicated in an ice bath for 1 hour. The mixture was then incubated by centrifugation at -20 °C for 1 hour, followed by centrifugation at 20,000 g for 15 minutes at 4°C. The supernatant was then incubated in a vacuum LVL-container for 1 hour. The supernatant was then harvested and dried under vacuum for LC-MS analysis. The samples were placed in an autosampler at 4°C throughout the analysis, and the samples were analyzed on a SHIMADZU-LC30 Ultra-High Performance Liquid Chromatography (UHPLC) system using an ACQUITY UPLC® HSS T3 (2.1 × 100 mm, 1.8 µm) (Waters, Milford, MA, USA) column. The injection volume was 6 μL, the column temperature was 40°C, and the flow rate was 0.3 mL/min. The chromatographic mobile phases were A: 0.1% formic acid aqueous solution and B: acetonitrile. The chromatographic gradient elution procedure was as follows: 0-2 minutes, 0 B; 2-6 minutes, B varied linearly from 0% to 48%; 6--10 minutes, B varied linearly from 48% to 100%; 10--12 minutes, B maintained at 100%; 12--12 minutes, B maintained at 100%; 12--12 minutes, B maintained at 100%; and 12--12 minutes, B varied linearly at 100%. B was maintained at 100%; 12 - 12.1 minutes, B changed linearly from 100% to 0%, and 12.1 - 15 minutes, B was maintained at 0%.

### 2.4 Hematoxylin-Eosin Staining of Rat Pancreas Tissue

The pancreas was removed from the rats, and the tissue was fixed in pre-portioned formalin EP tubes. The tissue was then processed for paraffin embedding and hematoxylin-eosin staining. Tissue sections were stained for β- cells, and sections were stained immunohistochemically using the aldehyde magenta-orange G special stain and insulin antibodies.

### 2.5 Statistical Analysis

All experimental data were statistically analyzed using SPSS 19.0, and the differences between multiple groups were significant. *p* ≤ 0.05 for a statistically significant difference.

## 3 RESULTS

### 3.1 Changes in Glucose Level in Rats

We compared the blood glucose of rats injected with Streptozotocin (STZ) at different time points with that of normal uninjected rats. We used fasting blood glucose above 11.1 mmol/L and random blood glucose above 16.7 mmol/L as criteria for successful modelling [16]. The blood glucose levels of rats injected with STZ tended to increase compared with those of uninjected rats under the same conditions. However, glucose-fed rats injected with STZ showed a smaller change in blood glucose, tentatively suggesting that glucose-fed rats were less affected by STZ-induced diabetes. All mannose-fed rats displayed elevated blood glucose levels after 48 hours, indicating that mannose may have a greater impact on blood glucose and that there may be a synergistic effect on STZ-induced diabetes (Fig. **1A**). It was evident that after injection of STZ under the same conditions, the success rate of hyperglycaemic modelling was significantly greater in the mannose group than in the fructose group, whereas the glucose group had the lowest modelling rate, and all had their blood glucose reduced to within the normal glycaemic range at 48 hours (Fig. **1B**).

### 3.2 Histopathologic Structure of the Rat Pancreas

#### 3.2.1 HE-Staining of Rat Pancreatic Islet Structure

We compared control rats fed different monosaccharides and found that glucose-fed rats had structurally intact islets, with clear borders and tightly arranged cells. The islet cells of rats fed fructose were irregularly arranged and oedematous. The islets of the rats fed mannose were disorganized, with cellular oedema and some inflammatory cell infiltration (Fig. **2A**-**D**).

We found that after STZ administration, the islet structure in normal rats was severely disrupted, with inflammatory cell infiltration, cellular disorganisation, and cellular oedema. The islets in the glucose group were more structurally intact, with cellular oedema and partial inflammatory cell infiltration. Rats in the fructose-fed group had disrupted islet structure and cellular oedema. Surprisingly, the islet structure in the mannose-fed group was severely damaged, with fusion of adjacent islets, unclear borders, and a large number of inflammatory cells infiltrating the islets (Fig. **2E**-**H**).

#### 3.2.2 Staining of Rat Pancreatic Islet Structural β-cells

The main cause of diabetes mellitus is the damage to pancreatic islet function, so we stained the pancreatic β-cells, which play a major role in the pancreatic islets, and observed that the three monosaccharides did not have a significant effect on the pancreatic β-cells of the rats, according to the staining of the pancreatic β-cells. After the rats were injected with STZ, the number of pancreatic β-cells in the glucose-fed and fructose-fed groups was higher than that in the normal-fed group, whereas the number of pancreatic β-cells in the mannose-fed group was similar to that in the normal-fed group (Fig. **3**).

#### 3.2.3 Immunohistochemical Staining of Rat Pancreatic Islet Structures for Insulin

It is well known that reduced insulin secretion is one of the main causes of diabetes mellitus. To explore this issue, we performed immunohistochemical staining of rat pancreatic islets for insulin. The results showed that the three monosaccharides had little effect on insulin antibody expression in rats. Notably, after receiving STZ injections, rats in the glucose- and fructose-fed groups showed a significant increase in insulin secretion and insulin antibody expression compared with those in the normal-fed group; however, no significant changes were seen in these two indices in the mannose-fed group (Fig. **4**).

### 3.3 Metabolomics Analysis of Rat Serum

#### 3.3.1 Data Quality Control

Partial Least Squares Discriminant Analysis (PLS-DA) uses partial least squares regression to model the relationship between metabolite expression and sample category to achieve the prediction of sample category. The closer R2Y and Q2 are to 1, the more stable and reliable the model is. The experimental results demonstrate the model's stability and the high quality and stability of the analytical method (Fig. **5**).

#### 3.3.2 Serum Differential Metabolites

Significantly different metabolites were identified using VIP > 1 and *p* < 0.05 as screening criteria, and their expression changes were analyzed. Comparing the serum differential metabolites in eight groups of rats, there were five differential metabolites in common possession, *i.e.*, Oxymetholone, Methoxyfenozide, FA 18:1+2O, Cevadine, and Camalexin (Fig. **6**, Table **1**). We analyzed the first 30 differential metabolites in each group during group discussion, and the changes in differential metabolites in rats after STZ injection are shown in Fig. (**7**).

Differential metabolites between control rats and rats injected with STZ (Control + STZ) were mainly characterized by the down-regulation of Rhamnose, Camalexin, and the up-regulation of Theobromine and 25S-Inokosterone (Fig. **7A**). Differential metabolites between glucose-fed rats (Glu) and glucose-fed rats after STZ injection (Glu + STZ) mainly showed down-regulation of Convoidine, Dimethachior Oxa, and up-regulation of 8-Deacetyl Yunaconitine, Pyrrolidine, and 25S-Inokosterone (Fig. **7B**). Differential metabolites between fructose-fed rats (Fru) and fructose-fed rats injected with STZ (Fru + STZ) were mainly characterized by the down-regulation of Pentadecanoyl Ethanolamide, L-Hyoscyamine, and the up-regulation of Creatine, Terbutylazine-Desethyl, and Tetradec-5-Ynoic Acid (Fig. **7C**). Differential metabolites between mannose-fed rats (Man) and mannose-fed rats after STZ injection (Man + STZ) were mainly characterized by the down-regulation of Cocamidopropylbetaine, Rhamnose, and L-Thyroxine, and the up-regulation of Tetradec-5-Ynoic Acid and Pantothenic Acid (Fig. **7D**).

#### 3.3.3 Analysis of Differential Metabolic Pathways

We carried out an exhaustive enrichment analysis of metabolic pathways based on metabolite differences, in-depth comparisons of potential key pathways of action across the different experimental groups, and elaborated clear bubble diagrams to highlight significant differences in metabolic pathways between the groups (Fig. **8**). We compared metabolic differences between several groups not injected with STZ and found a major focus on tyrosine metabolism, as indicated by enrichment for metabolic pathways, Pentose phosphate metabolism, and Type 2 diabetes mellitus (Fig. **8A**). The metabolic differential pathways in the injected STZ group consisted mainly of arginine and proline metabolism, the mTOR signalling pathway, and amoebiasis (Fig. **8B**).

#### 3.3.4 Differences in Serum Metabolites After Different Monosaccharide-Fed Rats

We analyzed the serum metabolic differentiators in rats given different monosaccharides and selected the top 10 VIP values for cluster analysis, as shown in Fig. (**9**). The main metabolites that were found to be significantly altered as a result of the study were Glycerate, Usnic Acid, Phakellistatin 13, Cephalochromin, Pentadecanoyl Ethanolamide, Sulfinpyrazone, and Procyanidin B2. There was a significant increase in Glycerate, Usnic Acid, Phakellistatin 13, Cephalochromin, N-Tigloylglycine, and 1-Palmitoyl-2-Oleoyl-Sn-Glycero-3-Phosphocholine. A decrease in Pentadecanoyl Ethanolamide, Sulfinpyrazone, Procyanidin B2, and L-Hyoscyamine in the serum of rats in the glucose, fructose, and mannose groups (Fig. **10**).

## 4 DISCUSSION

We compared the effects of different monosaccharides on blood metabolites in rats and observed significant changes in seven metabolites: Glycerate, Usnic Acid, Phakellistatin 13, Cephalochromin, Pentadecanoyl Ethanolamide, Sulfinpyrazone, and Procyanidin B2. These metabolites are mainly involved in glycolysis [17], cellular activity, and cytotoxicity pathways [18, 19]. Based on metabolite differential analysis, it was hypothesised that glucose, fructose and mannose may act on the rats through pathways mainly involved in Tyrosine metabolism, Pentose phosphate metabolism, and T2DM. Similarly, the analyses showed that the effects of Streptozotocin (STZ) in rats under different conditions were mainly mediated through the Arginine and proline metabolism, mTOR signaling pathway, and Amoebiasis.

Studies have shown that high sugar and fat intake can damage pancreatic β-cells, leading to Type 2 Diabetes Mellitus (T2DM); high sugar intake can trigger glucotoxicity by inducing cellular oxidative stress, further damaging β-cells [20, 21]. High intake of monosaccharides (especially fructose and glucose) is associated with the incidence of a variety of diseases, and their conversion by intestinal absorption is an important factor in the induction or exacerbation of immune disorders and inflammation [22, 23]. Dispersed islet cell populations in the pancreas regulate systemic glucose homeostasis, which is closely linked to systemic metabolic processes such as brain and lipid metabolism [24]. Excessive fructose intake has effects on liver metabolism [25], kidney metabolism [26] and intestinal metabolism [27], and fructose metabolism in the liver may be related to systemic amino acid metabolism, and changes in its associated enzymes may be correlated with insulin resistance [28] and glucose homeostasis [29]; Fructose intake plays a direct or indirect role in the development of chronic kidney disease and is capable of increasing the risk of kidney stones [30]. Fructose also induces intestinal and cardiovascular diseases, has an impact on the gut microbiome, imbalances the immune system, and induces the production of inflammatory factors [31]. In addition, fructose induces intestinal and cardiovascular diseases, contributes to immune imbalances, and promotes the production of inflammatory factors by altering the intestinal microbiota. Experimental evidence also suggests that fructose may promote tumour formation, metastasis, and cancer cell growth [32]. In contrast, mannose is primarily involved in the glycosylation of specific proteins and is metabolised to a limited extent *in vivo*, which has a lesser impact on blood glucose but confers anti-inflammatory and immunomodulatory effects [33, 34]. However, some studies have shown that mannose is an insulin-regulated metabolite, and its concentration in the blood is associated with the development of diabetes. Mannose levels are regulated by insulin and may serve as a potential marker for insulin resistance [35]. Therefore, we wanted to determine the effects of long-term intake of glucose, fructose, and mannose on the normal organism and whether they affect the prevalence of diabetes mellitus. The results of this experimental study show that long-term intake of low doses of fructose and glucose may reduce the risk of diabetes, and long-term intake of mannose has significant damage to the organism and may increase the risk of diabetes.

This study investigated the effects of glucose, fructose, and mannose on the pathological structure and serum metabolism of rat pancreatic islets. The results showed that glucose, fructose, and mannose exhibited different degrees of glycotoxicity in rats, with mannose exhibiting the greatest glycotoxicity, followed by fructose, and glucose exhibiting the least glycotoxicity. Analysis of histopathology and serum metabolites indicates that mannose, however, may induce β-cell damage and potentially contribute to T2DM development. These contrasting effects are likely mediated by the distinct interactions of each monosaccharide with pancreatic islet β-cells, mechanisms warranting further investigation.

## CONCLUSION

Glucose, fructose, and mannose are all hexose monosaccharides that are isomers of each other. They are structurally and metabolically closely related in living organisms, but also exhibit significant functional differences. Glucose is central to energy and blood glucose regulation, fructose is a highly efficient substrate for hepatic metabolism (excess is pathogenic), and mannose is an important protein-modifying molecule. They have differential and even opposing effects on pancreatic β-cell function. In this study, the dual potential roles of monosaccharide types in islet cell protection and damage were revealed by analysing the differential effects of three monosaccharides, glucose, fructose, and mannose, on rat pancreatic islet tissue function and serum metabolism. Notably, glucose exposure partially attenuated Streptozotocin (STZ)-induced pancreatic islet β-cell injury. Long-term intake experiments showed that all three types of monosaccharides produced progressive damage to islet function, with mannose having the most significant detrimental effect. This phenomenon may be related to the differences in the metabolic pathways of monosaccharides. Unlike glucose, which is efficiently metabolised through the glycolytic pathway, mannose must be phosphorylated to fructose-6-phosphate before entering metabolism, and its slow rate of metabolism may lead to a persistent high intracellular glucose state, resulting in hyperglycemic toxicity. The present study suggests that the metabolic characteristics of mannose may make it a risk factor for T2DM. Compared with fructose and glucose, the slow local metabolism of mannose in pancreatic islets is more likely to lead to microenvironmental hyperglycaemia, which may contribute to the development of T2DM through a dual mechanism.

## Figures and Tables

**Fig. (1):**
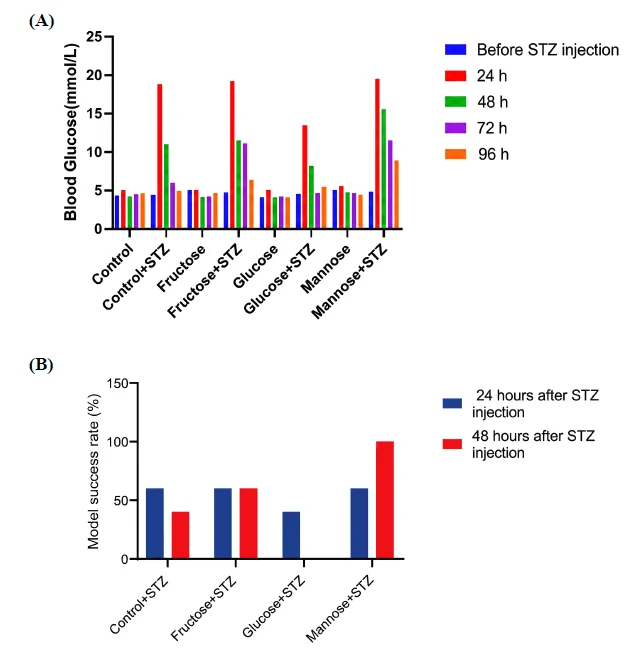
(**A**) Histograms of blood glucose changes in different groups of rats at 24, 48, 72, and 96 hours after STZ injection and before injection, and the Model success rate. (**B**) Modelling rates of diabetes in each group.

**Fig. (2):**
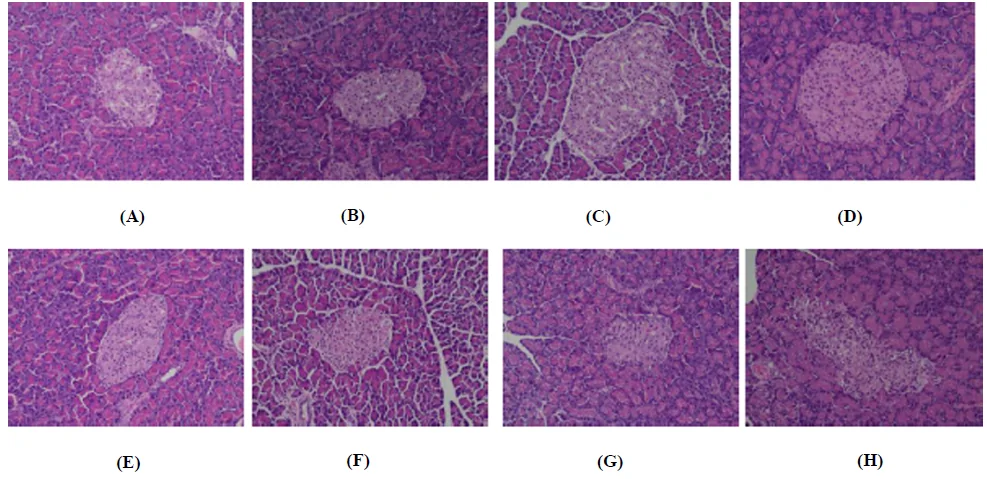
Histomorphometric analysis of rat pancreatic islet tissue (H&E,20X). (**A**) Control; (**B**) Glucose; (**C**) Fructose; (**D**) Mannose; (**E**) Control + STZ; (**F**) Glucose + STZ; (**G**) Fructose + STZ; (**H**) Mannose + STZ.

**Fig. (3):**
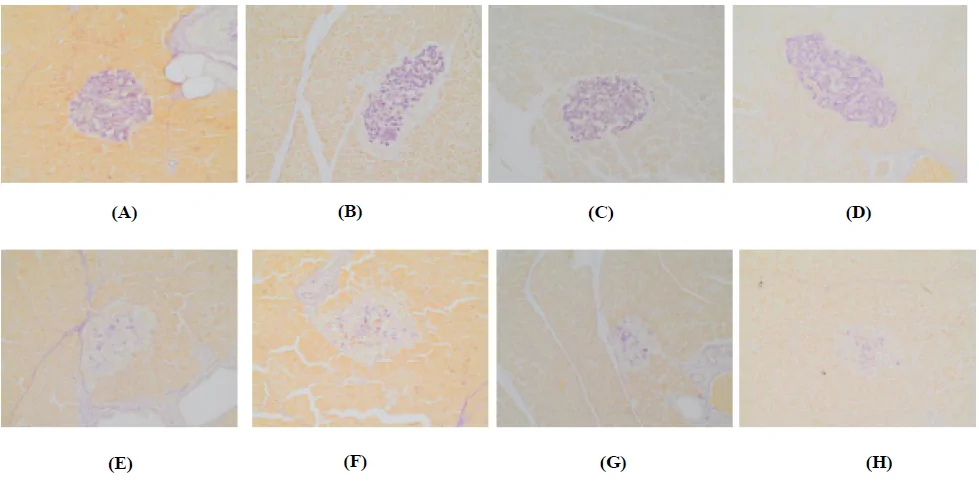
Histomorphometric analysis of rat pancreatic islet tissue (β cells staining, 20X). (**A**) Control; (**B**) Glucose; (**C)** Fructose; (**D**) Mannose; (**E**) Control + STZ; (**F**) Glucose + STZ; (**G**) Fructose + STZ; (**H**) Mannose + STZ.

**Fig. (4):**
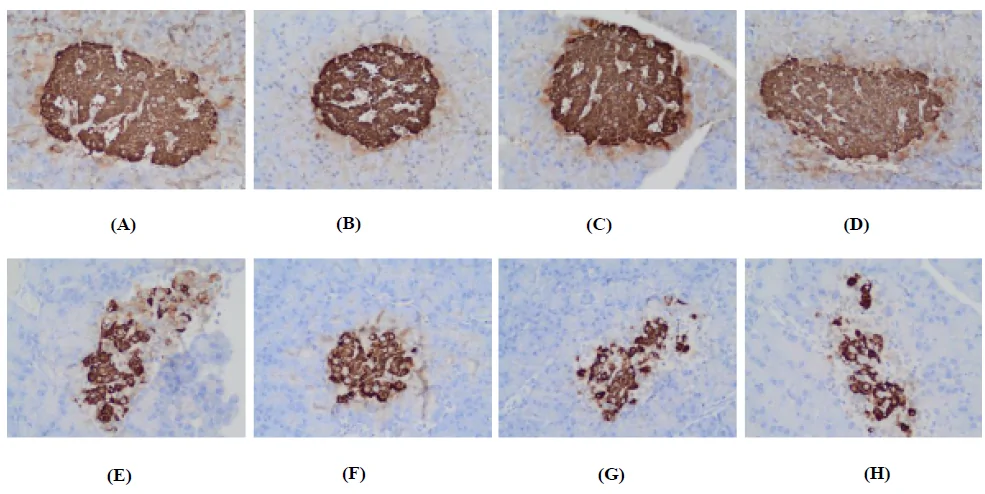
Histomorphometric analysis of rat pancreatic islet tissue (Immunohistochemical staining, 40X). (**A**) Control; (**B**) Glucose; (**C**) Fructose; (**D**) Mannose; (**E**) Control + STZ; (**F**): Glucose + STZ; (**G**) Fructose + STZ; (**H**) Mannose + STZ.

**Fig. (5):**
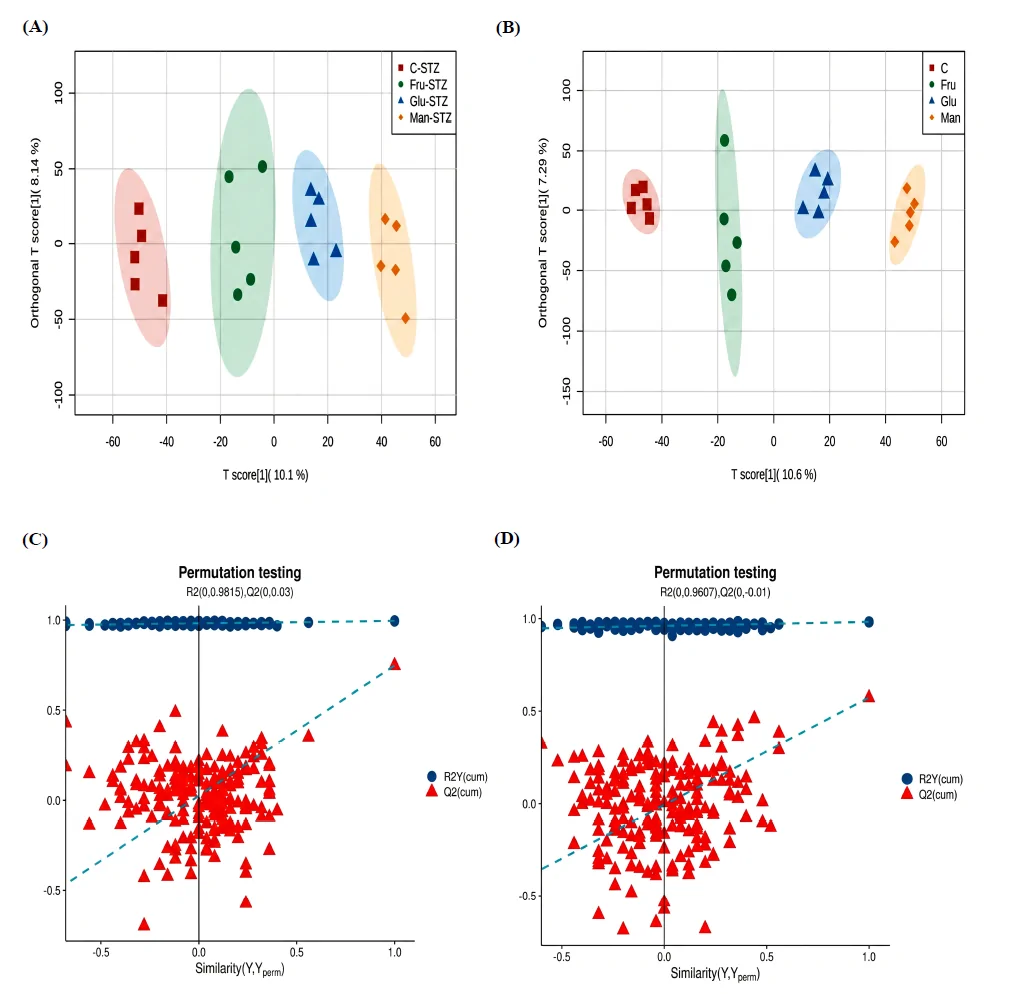
(**A, B**) PLS-DA; (**C**) Cross-validation plots for different monosaccharide groups; (**D**) Cross-validation plots of rat groups after STZ injections with different monosaccharides.

**Fig. (6):**
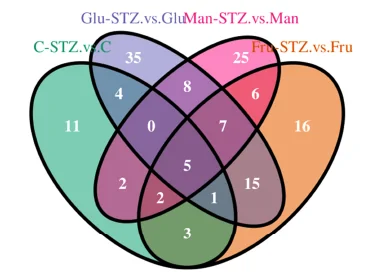
Venn diagram chart of rat serum metabolites.

**Fig. (7):**
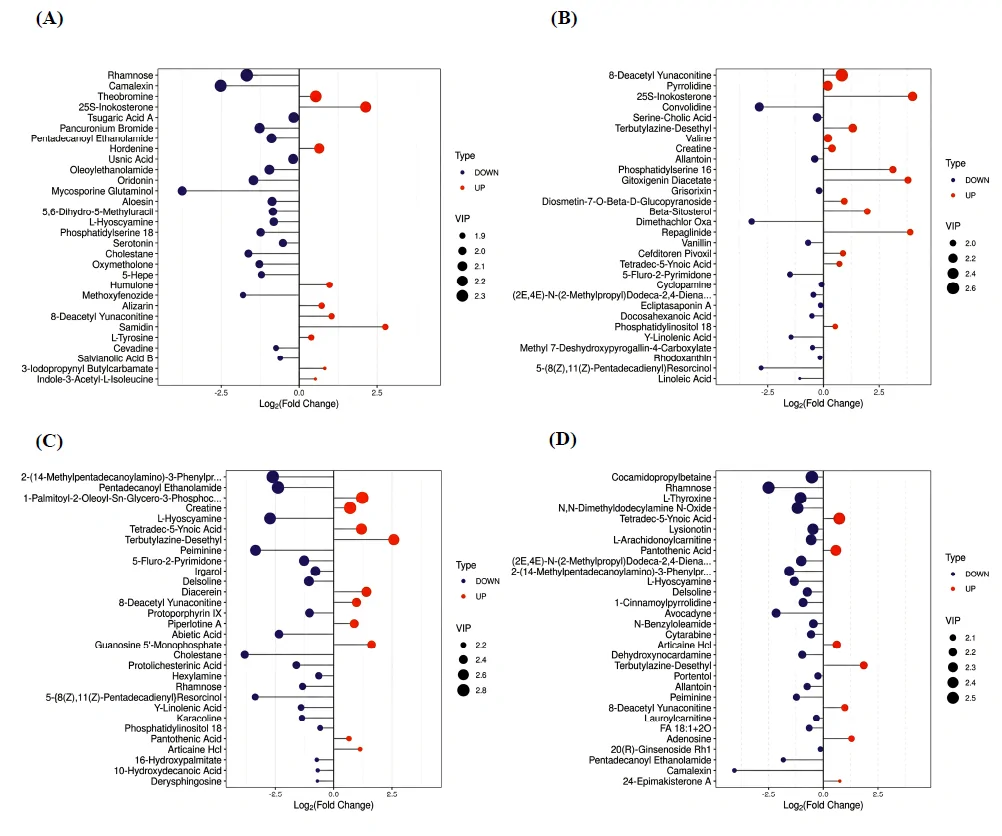
Schematic representation of the TOP 30 differential metabolites. (**A**) Control *vs.* Control + STZ; (**B**) Glu *vs.* Glu + STZ; (**C**) Fru *vs.* Fru + STZ; (**D**) Man *vs.* Man + STZ.

**Fig. (8):**
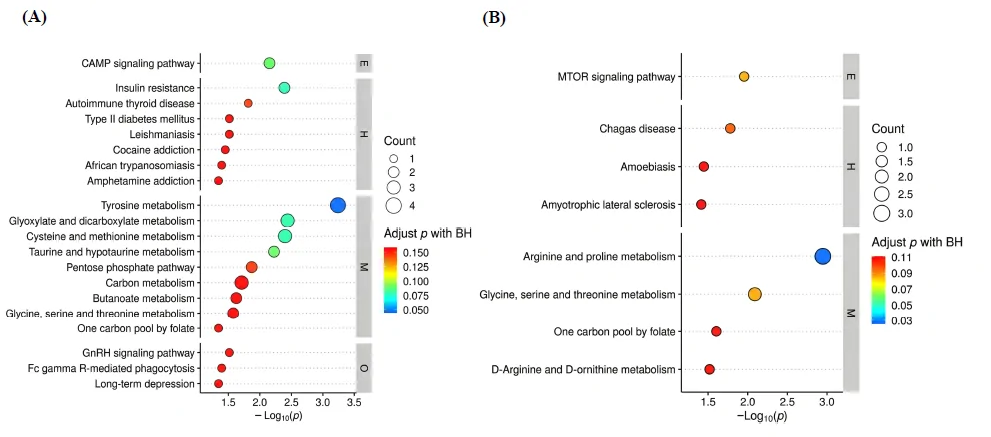
Schematic diagram of KEGG pathway bubbles. (**A**) Control *vs.* Glu *vs.* Fru *vs.* Man; (**B**) Control + STZ *vs.* Glu + STZ *vs.* Fru + STZ *vs.* Man + STZ).

**Fig. (9):**
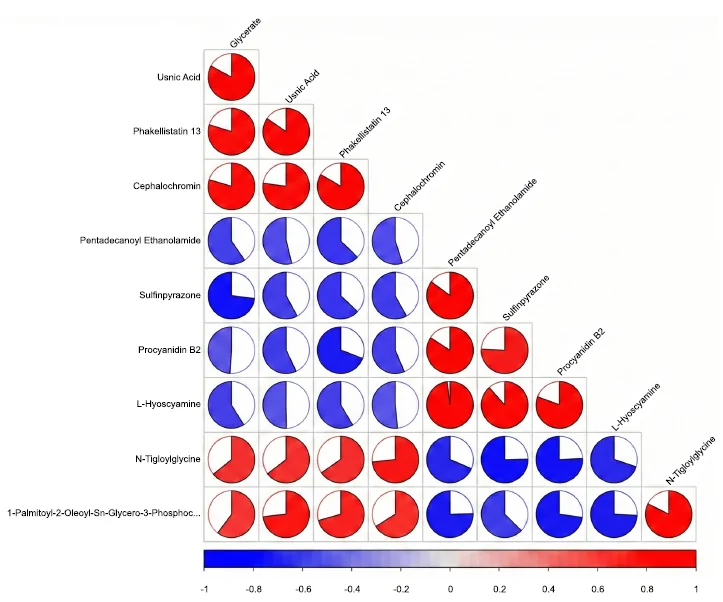
Heatmap of metabolite correlations of serum metabolite VIP top 10 differences in serum metabolites.

**Fig. (10):**
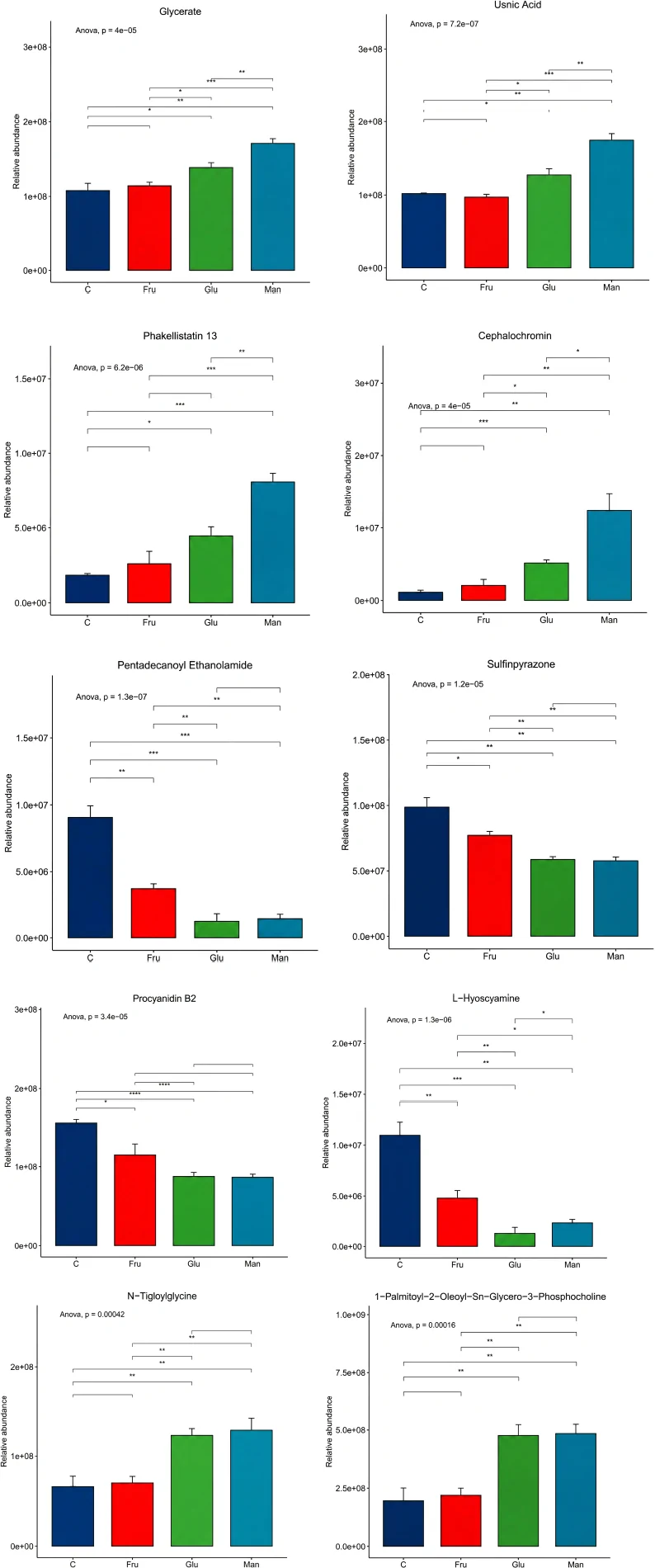
Bar chart of TOP10 changes in differential metabolites.

**Table 1:** Differential metabolites.

**MetaboName**	**Class**	**RT (min)**	**m/z**	**Formula**	***p*-value**	**VIP**
Methoxyfenozide	Benzene and substituted derivatives	7.084	367.20981	C_22_H_28_N_2_O_3_	0.0431	1.7983
Oxymetholone	Steroids and steroid derivatives	9.838	331.22528	C_21_H_32_O_3_	0.0064	1.9888
Camalexin	Indoles and derivatives	1.378	199.03572	C_26_H_44_O_9_	0.0218	1.7529
FA 18:1+2O	-	8.681	313.23596	C_18_H_34_O_4_	0.0031	1.8740
Cevadine	Lipids and lipid-like molecules	9.692	590.3421	C_32_H_49_NO_9_	0.0151	1.6782

## Data Availability

The data and supportive information are available within the article.

## LIST OF ABBREVIATIONS

STZ: Streptozotocin
IDF: International Diabetes Federation
T1DM: Type 1 Diabetes Mellitus
T2DM: Type 2 Diabetes Mellitus
GDM: Gestational Diabetes Mellitus
PLS-DA: Partial Least Squares Discriminant Analysis
